# In This Issue

**DOI:** 10.1002/cfg.344

**Published:** 2003-12


**Comparative and Functional Genomics**
*Comp Funct Genom* 2003; **4**: 569.
Published online in Wiley InterScience (www.interscience.wiley.com). **DOI:** 10.1002/cfg.344

### In this issue

### Microarray analysis of cardiac fibrosis of renal failure

Using microarrays to study a rat model of cardiac remodelling during renal failure, **Kerstin Amann** and colleagues observed patterns of gene activation pointing to signalling pathways involved in this process.

### A molecular phylogenomic analysis of the ILR1-like gene family

Using known members of this gene family from *Arabidopsis thaliana*, **James Campanella** and colleagues identified orthologues from other plant species and investigated the evolution and importance of this gene family in plants.

### Steady-state analysis of genetic regulatory networks

**Ilya Shmulevich** and co-workers analysed human glioma gene expression patterns using probabilistic Boolean networks (PBNs) to determine the joint steady-state probabilities for several groups of genes.

### Structure and evolution of the human fast skeletal troponin T gene

In sequencing this troponin gene, **Raymund Stefancsik** *et al.* have uncovered an exon–intron structure and regulatory motifs that shed light on the evolution of the gene and the origin of its developmentally regulated isoforms.

### Special section of selected reviews from the ESF Programme on Functional Genomics 1st European Conference, 'Functional Genomics and Disease 2003'

**Ian Dunham** gives us an overview of the characteristics of human genes, using data compiled from extensive study of human chromosome 22.
**Jiri Forejt** proposes the use of mice carrying a segmental trisomy of chromosome 17 to aid our understanding of Down's syndrome.
**Anne Cambon-Thomsen** discusses the ethical issues arising from large-scale biobanking for genomics, and how genomics can be used to improve biobanks.

### Special section of reports from the Intelligent Systems for Molecular Biology 2003 conference

**Catherine Abbott** reports on the keynote and prize presentations given at the main ISMB conference this year.
**Vincent Schachter** *et al.* bring us a report from the BioPathways special interest group (SIG) workshop.
**Phillip Lord** and **Robert Stevens** review the Bio-ontologies SIG workshop, which this year had a theme of 'From Text to Ontology and Back Again'.
**Christian Blaschke** and colleagues describe the talks and discussion topics raised at the Text Mining SIG.
**Christian Blaschke** *et al.* announce the launch of a competition for comparative assessment of text mining tools, BioCreAtIvE: Critical Assessment of Information Extraction systems in Biology.

### Conference Calendar

A listing of genomics-related conferences planned for March–May 2004.

Copyright © 2003 John Wiley & Sons, Ltd.

